# Modern Prodrug Design for Targeted Oral Drug Delivery

**DOI:** 10.3390/molecules191016489

**Published:** 2014-10-14

**Authors:** Arik Dahan, Ellen M. Zimmermann, Shimon Ben-Shabat

**Affiliations:** 1 Department of Clinical Pharmacology, School of Pharmacy, Faculty of Health Sciences, Ben-Gurion University of the Negev, Beer-Sheva 84105, Israel; 2 Department of Medicine, Division of Gastroenterology, University of Florida, Gainesville, FL 32608, USA

**Keywords:** molecular biopharmaceutics, membrane transporters, passive/active intestinal permeability, prodrug activation, targeted prodrug approach

## Abstract

The molecular information that became available over the past two decades significantly influenced the field of drug design and delivery at large, and the prodrug approach in particular. While the traditional prodrug approach was aimed at altering various physiochemical parameters, e.g., lipophilicity and charge state, the modern approach to prodrug design considers molecular/cellular factors, e.g., membrane influx/efflux transporters and cellular protein expression and distribution. This novel targeted-prodrug approach is aimed to exploit carrier-mediated transport for enhanced intestinal permeability, as well as specific enzymes to promote activation of the prodrug and liberation of the free parent drug. The purpose of this article is to provide a concise overview of this modern prodrug approach, with useful successful examples for its utilization. In the past the prodrug approach used to be viewed as a last option strategy, after all other possible solutions were exhausted; nowadays this is no longer the case, and in fact, the prodrug approach should be considered already in the very earliest development stages. Indeed, the prodrug approach becomes more and more popular and successful. A mechanistic prodrug design that aims to allow intestinal permeability by specific transporters, as well as activation by specific enzymes, may greatly improve the prodrug efficiency, and allow for novel oral treatment options.

## 1. Introduction

Prodrugs are derivatives of active drug moieties, designed to undergo conversion in the body, thereby releasing the active parent drug. The prodrug approach is taken in order to overcome pharmaceutical, pharmacokinetic, or pharmacodynamic obstacles, such as low oral absorption, inadequate site specificity, poor stability, *etc.* In recent years, prodrugs have become increasingly popular and successful; to date, ~10% of the global marketed medications are prodrugs, 20% of all small molecular medicines approved between 2000 and 2008 were prodrugs, and when focusing on 2008, it emerges that over 30% of drugs approved in this year were prodrugs [1,2,3].

During the past years, the pharmaceutical sciences have undergone a molecular revolution; no longer relying on empirical fitting based on plasma levels, the modern ADME (absorption, distribution, metabolism and excretion) research considers molecular/cellular factors, such as membrane transporters and cellular enzyme expression and distribution. This molecular revolution dramatically influenced today’s drug design and delivery at large, and the prodrug approach in particular, as will be presented in this article.

The prodrug approach is frequently utilized to increase drug absorption following oral administration. For that, the traditional/classic prodrug approach may be taken, e.g., to mask charged moieties and enhance drug lipophilicity and passive diffusion by various carboxylic acid esters, which release the active carboxylic acid after hydrolysis [4]. More recently, a novel “targeted-prodrug” approach has emerged thanks to the research of transporters and enzymes, utilizing carrier-mediated transport to increase drug absorption [5,6,7]. Naturally, this approach necessitates considerable understanding of the molecular and functional characteristics of the transporter.

In order to exert therapeutic effect, prodrugs must be activated to produce the active parent drug. This activation can be nonspecific; however, knowledge of the potential enzymes responsible for this process may help to rationale the design of successful prodrugs. By considering enzyme-substrate specificity, one may overcome inadequate site specificity, leading to higher efficacy and lower toxicity.

In this article, the concepts of modern *vs.* traditional prodrug approach will be presented. Examples for the two prodrug approaches, the traditional *vs.* the classical, will be offered. Finally, the new opportunities that the continuous molecular advancement in related fields brings to the field of orally administered prodrug will be discussed.

## 2. The Classical Prodrug Approach

Prodrugs or drug conjugates involve the synthesis of inactive drug derivatives that are converted to the active form in the body. The classical approach for prodrug design uses the non-specific strategy of covalently modifying the drug of interest by attaching hydrophilic functionalities (e.g., phosphate, sulfate) to increase the solubility of the parent drug [8,9,10], or lipophilic moieties (e.g., ester) to increase its passive permeability [11,12,13,14,15]. This approach lacks site specificity, and as a prodrug will eventually be converted into the active form [16]. The purpose for taking this approach is to overcome physicochemical or biopharmaceutical problems associated with the parent drug, hence providing an alternative approach to design less reactive and less cytotoxic form of drugs [17]. The objectives include altering the physicochemical properties of drugs to achieve modified drug pharmacokinetics, extended activity, reduced side effects, or increased selectivity. Specific aims include overcoming inadequate oral absorption, insufficient skin penetration, poor blood-brain barrier permeability, disruption in metabolic pathways and toxicity [18].

Following oral administration, free testosterone undergoes complete first pass metabolism, resulting in non-detectable systemic bioavailability. Testosterone undecanoate is a prodrug of testosterone esterified in the 17-position with undecanoic acid. During oral administration the majority of the prodrug is absorbed via the lymphatic system into the systemic circulation [19], and hence first pass hepatic metabolism is avoided [20,21,22]. More than 80% of the free testosterone detected in the plasma was contributed by hydrolysis of the lymphatically transported prodrug [23].

The development of topical and transdermal drug delivery systems aims to overcome the remarkably efficient barrier properties of human skin by employing non-toxic and non-irritant methods. For example, calcipotriol, Vitamin D_3_ derivative, has poor penetration through the stratum corneum into the epidermis which may explain its limited efficacy and frequent treatment failures in the treatment of psoriasis [24]. On the basis of increased lipophilicity prodrug design, new molecules that combine calcipotriol and poly-unsaturated-fatty-acids, through an ester bond, were synthesized. The ester bond was partially and gradually hydrolyzed by skin esterases, releasing the free active drug at high levels in the deep skin layers, leading to sustained drug delivery followed by prolonged activity [25,26].

Another challenging scope in drug delivery deals with poor blood-brain barrier drug permeability. Various drug delivery systems have been studied for delivery of pharmaceutical agents through the endothelial capillaries (BBB) for CNS therapeutics [27]. Enhanced brain delivery via prodrugs may be based on both the classical approach through increased lipophilicity and the modern approach which includes the utilization of transporters. Combining motives of both approaches represents a promising approach to enhance brain drug delivery via prodrug. In a recent study, the L-ascorbic acid prodrug of ibuprofen was synthesized and evaluated [28]. L-ascorbic acid has two bidirectional transporters in the brain; while the first one, GLUT1, leads to the entry into the brain, the second one, SVCT2, takes it outside of the brain. To avoid the L-ascorbic acid prodrugs from being evacuated out of the brain, a lipophilic thiamine disulfide system (TDS) was combined in order to help in two directions: to increase the lipophilicity of the drug (as per the classical prodrug approach) and to prevent the bidirectional active transport of the drug (as per the modern prodrug approach). The investigators concluded that this prodrug is a promising carrier system to enhance CNS drug delivery ability into brain [28].

## 3. The Modern Prodrug Approach

The molecular revolution has allowed for a modern prodrug approach to emerge, in which pro-moieties are covalently attached to the molecule of interest to selectively target certain transporters or enzymes [5,6,29]. This modern strategy offers a remarkable potential for improving drug bioavailability and selectivity of poorly absorbed drug molecules [30,31,32].

### 3.1. Targeting Transporters in Prodrug Design

The recent advances in biochemistry and molecular biology have provided a wide scope of information about the function and expression of transporters and enzymes. For instance, many transporters are expressed on both the apical and basolateral sides of the intestinal enterocytes, that may allow selective drug targeting. These include the organic anion transporters (OAT) family [33,34], organic cation transporter (OCT) family [35,36], sodium dependent bile acid transporter (ASBT) [37,38,39], sodium-dependent glucose transporter (SGLT) family [40], monocarboxylate transporter (MCT) family [41,42], amino acid transporter PAT1 [43,44], amino acid transporter ATB°^,+^ [45,46], folate transporter (PCFT) [47,48], and oligopeptide transporter (PEPT1) [49,50,51,52,53]. These transporters were characterized in detail, and have been shown to play important roles in the absorption of certain nutrients and drugs [54,55,56,57]. The information available on the substrate specificity of these transporters allows to chemically modify drug moieties so that their oral absorption will be enhanced via targeting intestinal transporters. PEPT1 has captured the greatest attention as a drug transport pathway, mainly due to wide distribution throughout the entire small intestine, broad substrate specificity and high capacity [53,58,59]. PEPT1 is characterized as a high-capacity low-affinity transporter, predominantly expressed in the small intestine, and accepts dipeptides, tripeptides, and peptidomimetic drugs such as β-lactam antibiotics and ACE inhibitors [51,60,61]. Thus, PEPT1 targeted prodrugs may present a promising strategy for enhanced oral drug delivery.

**Figure 1:**
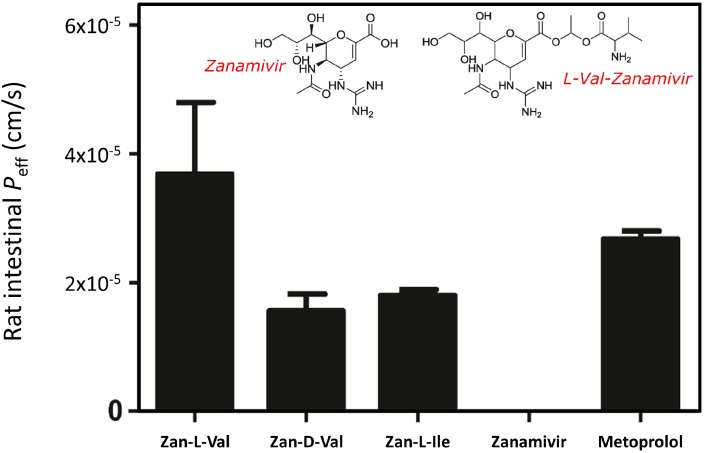
Molecular structure of zanamivir and its L-valyl prodrug, and the permeability of zanamivir and its amino acids prodrug in the single-pass rat jejunal perfusion method. Reproduced from [65] with permission.

FDA approved neuraminidase inhibitors for the treatment of influenza infection include two drugs, zanamivir and oseltamivir. Oseltamivir (tamiflu^®^) is a carboxylic acid ester that represents an example for the traditional prodrug approach; the low oral bioavailability (>5%) of oseltamivir carboxylate increased to ~80% for oseltamivir in humans [62]. While no reports indicating resistance against zanamivir could be found, there are several reports in the literature on resistance to oseltamivir [63]. However, the polarity of zanamivir results in extremely low oral bioavailability (~2%) [64]. The modern approach for prodrug design was taken in attempts to improve the oral absorption of zanamivir, targeting PEPT1 for transporter-mediated absorption [65]. A series of acyloxy ester prodrugs of zanamivir conjugated with amino acids were synthesized and characterized for chemical stability, membrane transport, and enzymatic activation. The L-valyl prodrug of zanamivir showed three-fold higher uptake by PEPT1 overexpressing cells in comparison to zanamivir, indicating recognition between the prodrug and the transporter. Intestinal permeability studies of amino acid zanamivir’s prodrugs compared to the parent drug confirmed that this targeted prodrug strategy greatly improved zanamivir's intestinal permeability (Figure 1) [65]. This research demonstrates that the modern prodrug approach has significant potential to allow for oral zanamivir therapy, and since no oral product of zanamivir currently exists [66], this may represent new treatment options in fighting the common seasonal flu as well as the recent H1N1 global pandemic [67].

A variety of amino acid, dipeptide and tripeptide prodrugs have been investigated in recent years for their suitability as PEPT1 substrates, including the anticancer drug floxuridine [68,69,70,71], the antiviral drugs acyclovir, gancyclovir, and zidovudine [72,73], the anticancer drugs melphalan [74] and gemcitabine [75,76], the antihypotensive agent midodrine [77], the antiosteoporotic drug alendronate [78,79], and more. Structure–activity relationship studies have assured that mono amino acid and dipeptide ester prodrugs generally provide enhanced PEPT1-mediated transport, resulting in improved oral absorption and bioavailability.

**Figure 2:**
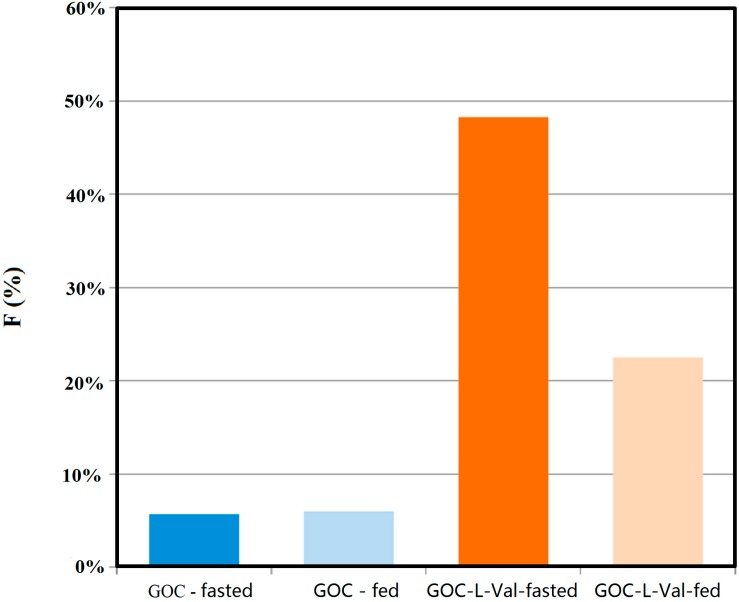
Systemic oral bioavailability of GOCarb and its L-Valine prodrug following oral administration of 10 mk/kg (*n* = 5). Reproduced from [80] with permission.

Another recently published study investigated the highly active polar antiviral agent, guanidine oseltamivir carboxylate, which has very low oral bioavailability (4%) [80]. In order to enhance its oral bioavailability, a series of acyloxy(alkyl) ester prodrugs conjugated with amino acids were synthesized and characterized. The results indicated 2–5-fold increased intestinal permeability, as well as ~30-fold increased affinity for PEPT1, of the amino acid prodrugs compared to the parent drug, demonstrating high recognition between the prodrugs and the transporter. The most promising derivative was found to be the guanidine oseltamivir carboxylate-L-Val, with 28% systemic oral bioavailability in mice under fed conditions, and 48% under fasted conditions, while the parent drug exhibited bioavailability of only 5% under both fed and fasted state (Figure 2) [80].

Targeting of monocarboxylate transporter type 1 (MCT1) has also been shown to enhance intestinal prodrug/drug absorption. This low-affinity high-capacity transporter accepts unbranched aliphatic monocarboxylates, and is widely expressed along the entire intestinal tract [81]. The GABA analogue gabapentin suffers from many poor PK properties (high variability, saturable absorption, lack of dose proportionality), and hence XP13512, a carbamate prodrug of gabapentin, was synthesized. The systemic bioavailability of gabapentin was dramatically increased after oral administration of XP13512, in both preclinical [82] and clinical [83] studies, while MCT1 plays a significant role in this absorption enhancement [82,84].

The sodium dependent bile acid transporter (ASBT) was also investigated as a potential prodrug target [37,85], and significantly improved absorption of the antiviral agent acyclovir [86] and the GABA analogue gabapentin [87] have been reported. Other transporters have also been targeted, as described above. Overall, this modern approach for oral prodrug design represents a mechanistic and intelligent strategy to increase oral absorption of poorly permeable compounds. Since intestinal permeability is, alongside the drug solubility, the most important factors dictating drug absorption following oral administration [88,89,90,91,92,93], the improved permeability achieved by this approach may enhance “developability” and drug-like properties [94,95,96], thus allowing new oral treatment options.

In a unique recent study, the modern prodrug approach was taken in order to avoid recognition between the protease inhibitor agent lopinavir and the efflux transporters P-gp and MRP2, which together with extensive metabolism by CYP3A4, limit the drug’s absorption. Three amino acid prodrugs of lopinavir were designed and investigated for their potential to circumvent efflux processes and also first pass metabolism [97]. Indeed, these prodrugs were shown to carry significantly lower affinity towards P-gp and MRP2 relative to the parent drug lopinavir. Moreover, the prodrugs exhibited higher liver microsomal stability relative to the parent drug lopinavir, suggesting circumvention of first pass metabolism [97]. This study demonstrates that the modern prodrug approach can be used not only to target desired transporters, but also to circumvent undesired processes and thereby enhance “developability” and drug-like properties.

### 3.2. Targeting Enzymes in Prodrug Design

An essential step in effective prodrug therapy is the activation of the prodrug and the release of the free active therapeutic agent. Important enzymes involved in the activation and bioconversion of ester-based prodrugs include paraoxonase, carboxylesterase, acetylcholinesterase, and cholinesterase [98,99,100]. Previously, the mechanism of the prodrug activation was often overlooked, and as long as the free parent drug was liberated, the activation was considered successful, and the activating enzymes did not draw much attention. In recent years, however, it is well recognized that substantial knowledge of the activating enzyme/s can aid to rationally design successful prodrugs. Hence, identifying the activating enzymes of different prodrugs can provide significant new targets for the design of effective therapeutic agents.

Valacyclovir is the 5'-valyl ester prodrug of the antiviral drug acyclovir. Valacyclovir increased the oral bioavailability of its parent drug acyclovir by 3- to 5-fold [101], attributable to carrier-mediated intestinal transport of the prodrug by PEPT1 [53,72,102]. Hence, valacyclovir is a successful example for the modern transporter-targeted approach for prodrug design. However, valacyclovir’s efficiency as an antiviral agent relies also on the rapid conversion of the prodrug to acyclovir *in vivo*. At first, it has been shown that enzymatic (rather than chemical) hydrolysis is the predominant activation mechanism of valacyclovir [103,104,105]. Then, it was found that valacyclovir is relatively stable in the gastrointestinal milieu, but carries high susceptibility to intracellular enzymatic hydrolysis [106]. Also, several peptide fragments of the major activating polypeptide were purified and sequenced [107]. Only in 2003, Kim *et al.*, succeeded to purify, identify and characterize the enzyme that activates valacyclovir to acyclovir in humans, naming it valacyclovirase, a serine hydrolase containing a catalytic triad S122-H255-D227 [108,109]. It was found that valacyclovirase is one of the main enzymes activating amino acid ester prodrug with high specificity, attributed to the critical residue D123 forming electrostatic interaction with the α-amino group of substrates. Valacyclovirase contains a large leaving group accommodating groove, which accommodates a variety of leaving groups, e.g., nucleoside analogues, as well as simple alcohols such as methanol, ethanol, and benzyl alcohol [98,110,111,112,113].

**Figure 3:**
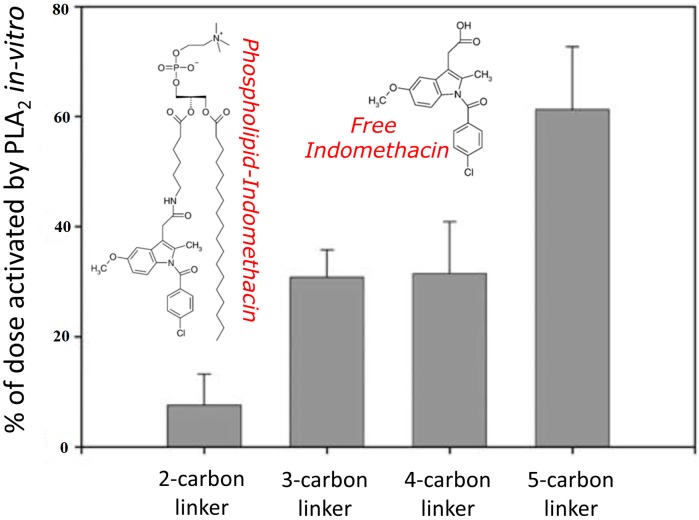
Activation of a series of phospholipid-indomethacin prodrugs by PLA_2_ enzyme *in vitro*. Reproduced from [12] with permission.

A novel type of prodrug was designed and synthesized, conjugating the non-steroidal anti-inflammatory drug indomethacin in place of the *sn-2* positioned fatty acid of a phospholipid, through a linker [12,13,114,115,116]. Physiologically, the fatty acid in the *sn-2* position is liberated by the enzyme phospholipase A_2_ (PLA_2_), resulting in a free fatty acid and a lysophospholipid as the lipolysis products [117]. The substitution of the *sn-2* positioned fatty acid by a drug moiety, hence, was designed to target PLA_2_ as the activating enzyme for this prodrug. Although it was reported that PLA_2_ strictly requires a fatty acid at the *sn-2* positioned [118], we have found that, depending on the carbonic linker length, PLA_2_ was able to recognize and activate the phospholipidic prodrugs of indomethacin both *in vitro* (Figure 3) and *in vivo*. A follow up study in rats revealed that after oral administration there was no absorption of the intact prodrug, however, the prodrug was continuously activated by PLA_2_ throughout the entire intestinal tract, resulting in a controlled release profile of the liberated free indomethacin in the systemic circulation [12]. This research illustrates the advantage of rational activating-enzyme targeted prodrug design, which minimizes the empirical elements of the activation process, and allows to better control the liberation of the free active drug moiety.

**Figure 4:**
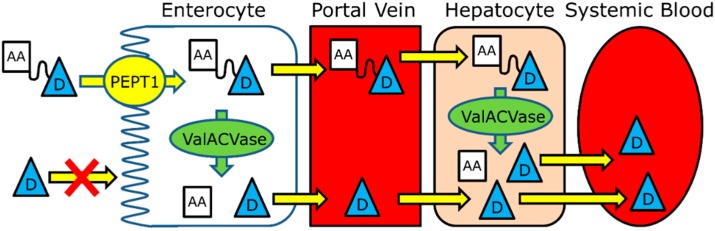
Illustration of the ‘double targeted’ prodrug approach, accounting for both transport via PEPT1 and activation via valacyclovirase.

While the amino acid ester prodrug strategy was applied to many nucleoside analogues and was successful in improving PEPT1-mediated oral absorption, the activation of these prodrugs was not well-studied and was assumed to be nonspecific until the identification of valacyclovirase. A unique modern approach to prodrug design, in which both transport and activation processes are accounted for already at the initial design stage, was taken (Figure 4). In this double-targeted prodrug approach, a series of amino acid esters of a model guanidine containing compound, 3-HPG, was synthesized and evaluated for both transport and activation [113,119,120]. Valine and isoleucine esters of 3-HPG had a significantly higher intestinal permeability than the parent compound, attributable to PEPT1-mediated transport. Remarkably, these L-amino acid prodrugs of 3-HPG were shown to be effectively activated by valacyclovirase, with *K*_m_ values in the range of the positive control valacyclovir, thereby liberating the parent moiety [119]. This novel approach, in which both transport and activation are taken into consideration already at the earliest design stages, represents the next step in the modern approach to drug delivery using prodrugs. This mechanistic approach may greatly minimize the empirical elements of the development and hence may result in better products with more predictable performance compared to the traditional practice.

## 4. Conclusions

Previously, the prodrug approach was viewed as a last option strategy, after all other possible solutions were exhausted; this is no longer the case. In fact, the prodrug approach should be considered already in the very earliest development stages. Indeed, the prodrug approach becomes more and more popular and successful.

The molecular revolution has significantly changed the pharmaceutical sciences in general, and the way we use the prodrug approach in particular. While the traditional approach was aimed at altering various physiochemical parameters, e.g., lipophilicity and charge state, the modern prodrug approach considers molecular/cellular factors, e.g., membrane influx/efflux transporters and cellular protein expression and distribution [121,122,123]. A mechanistic design that aims to allow intestinal permeability by specific transporters, as well as activation by specific enzymes, may greatly improve the prodrug efficiency, and allow for novel oral treatment options. Minimizing the empirical elements by taking the targeted prodrug approach represents an intelligent and powerful process, as the outcomes may be significantly more predictable; knowledge of the activating enzyme(s) may potentially speed the prodrug development process and lower its cost. Additionally, good knowledge of the transporter(s) and enzyme(s) involved in the absorption and activation may allow to predict and recognize potential competition-based drug-drug interactions [124,125]. A critical aspect that was not covered in this paper is the site-specific targeting potential; a prodrug designed to be activated by a specific enzyme that is overexpressed in the target site may allow to target the free drug to the site of action, resulting in improved efficacy and reduced toxicity.

Overall, in the near future, more information will certainly become available regarding transporters and enzymes that may be exploited for the targeted modern prodrug approach. Awareness of such information opens promising opportunities for precise and efficient drug delivery, as well as enhancement of treatment options and therapeutic efficacy.
